# Ongoing Assessment of the Molecular Evolution of Peste Des Petits Ruminants Virus Continues to Question Viral Origins

**DOI:** 10.3390/v13112144

**Published:** 2021-10-25

**Authors:** Mana Mahapatra, Richa Pachauri, Saravanan Subramaniam, Ashley C. Banyard, Shanmugam ChandraSekar, Muthannan Andavar Ramakrishnan, Felix Njeumi, Dhanavelu Muthuchelvan, Satya Parida

**Affiliations:** 1 The Pirbright Institute, Ash Road, Pirbright, Surrey GU24 ONF, UK; mana.mahapatra@pirbright.ac.uk; 2 Division of Virology, ICAR-Indian Veterinary Research Institute, Mukteswar, Nainital, Uttarakhand 263 138, India; richapachauri.1@gmail.com (R.P.); schand_vet@yahoo.co.in (S.C.); maramakrishnan@gmail.com (M.A.R.); 3 Directorate of Foot and Mouth Disease, National FMD Seromonitoring Laboratory, IVRI Campus, Bengaluru 560024, India; s.subramaniam@icar.gov.in; 4 Animal and Plant Health Agency (APHA), Woodham Lane, Addlestone KT15 3NB, UK; Ashley.Banyard@apha.gov.uk; 5 Food and Agriculture Organization of the United Nations (FAO), Viale delle Terme di Caracalla, 00153 Rome, Italy; Felix.Njeumi@fao.org

**Keywords:** peste des petits ruminants virus, molecular epidemiology, evolution, lineage divergence, full genome

## Abstract

Understanding the evolution of viral pathogens is critical to being able to define how viruses emerge within different landscapes. Host susceptibility, which is spread between different species and is a contributing factor to the subsequent epidemiology of a disease, is defined by virus detection and subsequent characterization. Peste des petits ruminants virus is a plague of small ruminant species that is a considerable burden to the development of sustainable agriculture across Africa and much of Asia. The virus has also had a significant impact on populations of endangered species in recent years, highlighting its significance as a pathogen of high concern across different regions of the globe. Here, we have re-evaluated the molecular evolution of this virus using novel genetic data to try and further resolve the molecular epidemiology of this disease. Viral isolates are genetically characterized into four lineages (I−IV), and the historic origin of these lineages is of considerable interest to the molecular evolution of the virus. Our re-evaluation of viral emergence using novel genome sequences has demonstrated that lineages I, II and IV likely originated in West Africa, in Senegal (I) and Nigeria (II and IV). Lineage III sequences predicted emergence in either East Africa (Ethiopia) or in the Arabian Peninsula (Oman and/or the United Arab Emirates), with a paucity of data precluding a more refined interpretation. Continual refinements of evolutionary emergence, following the generation of new data, is key to both understanding viral evolution from a historic perspective and informing on the ongoing genetic emergence of this virus.

## 1. Introduction

Peste des petits ruminants (PPR) constitutes a significant burden to sustainable agriculture across endemic areas, currently limited to Africa and Asia [1,2]. PPR is a highly contagious viral disease that primarily affects sheep and goat populations across endemic areas. Infection with PPR virus (PPRV) can lead to variable clinical disease outcomes, and whilst the virus can circulate, causing only mild disease within some populations, it is often associated with explosive outbreaks of severe disease, causing high morbidity and mortality rates during epizootics [2].

PPR is caused by the infectious viral agent PPRV, which is a non-segmented negative-strand RNA virus that has been phylogenetically classified, initially by partial sequence analyses and later through full genome analysis, into four distinct genetic lineages (I−IV) [3]. Lineage I contains viruses from West Africa, with very few defined isolates that were believed to have become extinct; however, recent studies have suggested that lineage I PPRV persists in West Africa, despite the emergence of apparently more dominant lineages [4]. Lineage II is also of West African origin, with historic isolates being described from Nigeria and Benin, with more contemporary detections also being from West Africa. Historically, lineage III consists of viruses detected in both the Arabian Peninsula and East Africa, although more recent isolates have been described in East Africa. Finally, lineage IV contains isolates that historically have an origin in Central Africa, but, for a long period of time, have been detected across Asia, in particular, India. Lineage IV viruses have been proposed as becoming dominant across endemic areas, and although poorly understood, this factor is important when considering viral evolution.

The success of the global rinderpest virus (RPV) eradication program, a closely related viral agent that primarily affected bovids, was sufficient to stimulate interest in PPRV [5]. The linkage was clear, as nearly all aspects of rinderpest virology and biology are closely related to those of PPRV, with only the primary host (large ruminants for RPV; small ruminants for PPRV) differing. Indeed, even the vaccinology behind protecting animals against RPV and PPRV is very similar, with live attenuated vaccines for both rinderpest and PPR being available for over 70 years and 30 years, respectively [6,7]. The renewed interest in PPRV, following the successful global eradication of RPV, has led to a global eradication program targeting PPR, with the end goal being achieved by 2030 [8].

Highly effective vaccines against PPRV have been available for over 30 years, and these vaccines are able to induce sterilizing immunity in the majority of cases where a vaccine of high quality is administered in a timely manner across animal populations. However, the species susceptibility to PPRV infection is very poorly understood, with myriad host and viral factors likely influencing the outcome of infection. To date, a broad range of domestic and wild species within the order Artiodactyl have been associated with infection, through either the detection of an active infection or previous exposure following antibody detection [2]. Alongside the significant impact on domesticated livestock, where the economic impact is most significant, PPR has also been associated with infection of zoological collections [9] as well as numerous endangered species, where infection has had a devastating impact on populations across different species [10,11]. From a conservation perspective, the decimation of the critically endangered Mongolian saiga antelope (Saiga tatarica mongolica) population, during 2016–2017, again raised the profile of this important infectious disease [11,12].

Despite the growing knowledge of PPRV and its distribution across endemic areas, the evolution of the virus is unclear, and many fundamental features of both viral and host interactions remain undefined. The paucity of full sequence data from different regions has led to contrasting outputs for the emergence of PPR in different regions. Considering that the first full genome for PPRV was not available until 2005 [13], a significant retrospective analysis of samples was required. As tools to develop full genome sequences have been developed, they have been further applied to historic samples. Initial studies looking at full genome assessment and the evolution of PPRV indicated that the virus emerged early in the 20th century [14], and this aligns with the formal recognition of the virus as a disease entity in the 1940s [15]. The evolution of different lineages has remained a significant question, with studies differing in opinion on the likely emergence of different lineages [14,16,17]. From an eradication perspective, whilst it is not predicted that the virus will evolve to escape post-vaccinal immune responses, it is critical to monitor viral emergence and assess sequence data of viruses that are emerging in different regions. Here, we analyze a wider data set, incorporating new full genomes from PPR-infected animals, and look across the global data set to evaluate factors involved in the evolution of this virus. The continual re-assessment of the evolutionary relationships across PPRV lineages is important with the generation and accessibility of new data.

## 2. Materials and Methods

### 2.1. Sample Collection and Whole Genome NGS

The complete genome sequences of two PPRV isolates from India were determined in this study. These viruses were isolated from archived suspected tissue samples of goats collected during two different PPR outbreaks from Himachal Pradesh. One outbreak occurred during 1999 in Chirgaon village of Shimla District (PPRV/IND/HP/Chirgaon/99) and the other in 2016 in Palampur (PPRV/IND/HP/Palampur/16). The whole genome sequencing was carried out by commercial NGS following the method as described by Masdooq and colleagues [18].

### 2.2. Data Set Curation

In addition to the two novel PPRV complete genome sequences generated in this study, all full genome sequences of PPRV (*n* = 94) available in Genbank (accessed on 1 September 2021) and 18 Israel full genome sequences of Clarke and colleagues [19] were included in the data set, making it a total of 114 sequences. However, 11 sequences with vaccine/reference strain sequences (highly passaged), incomplete sequences and sequences with recombination potential were excluded as these sequences could adversely bias outputs. This left a total of 103 sequences for subsequent analysis (Supplementary Table S1). Our data set contained sequences from 25 countries and had 24 more complete genome sequences compared to the previous analyses reported to date. Lineage IV dominated this data set with 85 sequences followed by lineage II (*n* = 9) and lineage III (*n* = 7). However, lineage I had the least, with only two sequences indicating continual paucity of full genome sequence data for lineages I−III (Supplementary Table S1). Within lineage IV, PPRV genome sequences from China were the most (*n* = 38).

### 2.3. Model Selection and Phylogenetic Analysis

A total of eight models were tested using the software jModeltest 2.1.10 [20] to select a statistically appropriate model to study the evolution of PPRV. Of these, the DNA substitution model GTR + G + I best suited our data set and was used subsequently for phylogenetic and evolutionary analyses. With a guide tree derived from Clustal X and asynchronous tip ages, TempEst was used to test the temporal signal and clock-likeness of the PPRV data set [21]. The evolutionary rate, sampling times, and change in effective population size were estimated using the Bayesian Markov chain Monte Carlo (MCMC) approach implemented in BEAST v 1.10.4 [22]. Using marginal likelihoods estimated by path sampling (PS) and stepping-stone (SS) sampling, the best-fit clock model (among strict clock, and relaxed clock with lognormal and exponential distribution) and the best-fit tree prior (among the constant size, exponential growth, Bayesian skyline coalescent, and Gaussian Markov random field (GMRF) Bayesian skyride) were chosen [23]. A uncorrelated exponential relaxed clock (UCED) was preferred over a strict clock and an uncorrelated lognormal relaxed clock (UCLD). Furthermore, the data set exhibited a coefficient of variation of 0.972, indicating significant rate of heterogeneity among branches and bolstering the case for using a relaxed clock. Constant size provided the greatest match for our data set among the coalescent tree priors under UCED, and it was chosen for evolutionary and demographic research.

To find root state probabilities, a discrete phylogeographic analysis was performed using a typical continuous-time Markov chain (CTMC) model with Bayesian stochastic search variable selection (BSSVS). To estimate changes in the effective population size over time, the non-parametric coalescent-based Bayesian skyline coalescent model (BS) was utilized. MCMC sampling was run for 4 × 10^8^ generations, with trees and posteriors sampled every 10^4^ steps. Two separate runs were completed and combined. Tracer 1.7 was used to visually check the convergence of all parameters. Tree Annotator was used to summarize the posterior tree distributions. The 95 percent highest probability density (HPD) values reflect statistical uncertainties in the data. The MCC consensus tree was plotted using FigTree version 1.4.4. Bayesian Tip-association Significance testing (BaTS) was used to assess the statistical significance of relationships between phylogeny and geographic location [24]. Based on the posterior samples of trees constructed by MrBayes 3.1, the association index (AI), parsimony score (PS), and monophyletic clade (MC) size were determined [25]. Each statistic’s null distribution was determined using 1000 random permutations.

## 3. Results and Discussion

### 3.1. PPRV Genome Sequences of New Indian Isolates

The two full genome sequences generated in this study have been submitted to GenBank under the accession numbers MN920706 and MN920707. Both the Indian isolates studied in this investigation (PPRV/IND/HP/Chirgaon/99 and PPRV/IND/HP/Palampur/16) were found to belong to lineage IV. Of these, the Chirgaon isolate (PPRV/IND/HP/Chirgaon/99; accession no.: MN920706) had a complete genome of 15,948 nucleotides, whereas the second isolate (PPRV/IND/HP/Palampur/16; accession no.: MN920707) had a deletion of six nucleotides in the M−F intergenic region (4487–4492). The six-nucleotide deletion in the later isolate was confirmed by RT-PCR, followed by Sanger sequencing. This deletion was also reported previously in another Indian isolate [18].

### 3.2. Evolutionary Rates and Lineage Divergence

**Figure 1:**
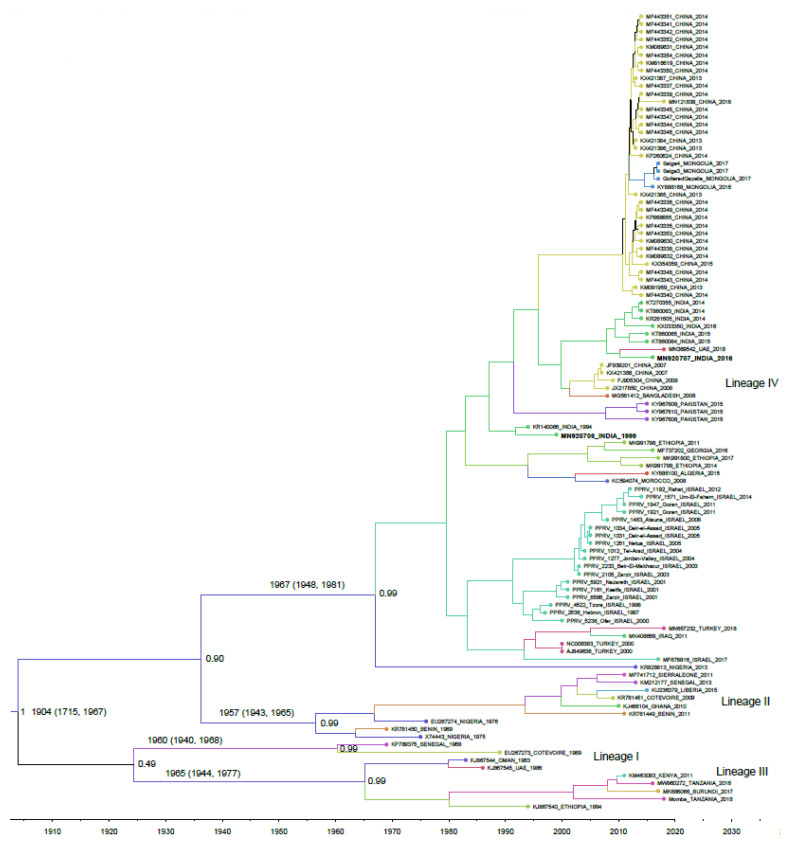
Bayesian maximum clade credibility phylogenetic tree based on the whole genome sequences of 103 PPRVs. Branch lengths are scaled in units of time, as indicated by the time axis. Branch colors denote inferred location. The node age and 95% HPD intervals are indicated. Posterior probabilities for major nodes are shown. Isolates sequenced in this study are shown in bold face.

A regression of root-to-tip distances versus sampling dates was calculated using TempEst. The slope of the regression (representing the rate) was 5.0229 × 10^−4^, with a correlation coefficient (variation in rate) of 0.8944, R-squared value of 0.7999, and residual mean squared value of 6.6524 × 10^−6^, indicating a stronger temporal signal in the data set for molecular clock analysis. The PPRV isolates were categorized into four distinct lineages (I−IV) in the maximum clade credibility (MCC) tree, as determined in previous investigations (Figure 1). The Indian isolates studied in this investigation were found to be divided into two sub-clades within lineage IV. One sub-clade received 99 percent posterior probability support and included two samples that were acquired in 1994 and 1999. The other clade contained isolates collected in India between 2014 and 2016, as well as one isolate collected in the UAE in 2018, and was also supported by 99% posterior probability.

The TMRCA estimate obtained from the 103 PPRV set indicates that PPRV possibly emerged in 1904 (95% HPD 1715–1968) (Figure 1). The estimate of the analysis is directly comparable to that reported earlier (1904, 95% HPD 1730–1966) [14]. However, the TMRCA estimate is somewhat older than that reported recently (1919, 95% HPD 1884–1945) [17], and is younger than the following previous reports: 1898, 95% HPD 1691–1945 [26]; 1870, 95% HPD 1691–1945 [19]. It is worth noting that estimates can be influenced by the number and length of the sequences used in analyses. Collectively, it can be speculated that the currently circulating PPRV might have originated during the late 19th century to early 20th century, before it was first documented in 1942 in Cote d’Ivoire. The node age of the common ancestor of lineage II and IV was estimated to be 1909, which is slightly older than that for the common ancestor of lineages I and III (Table 1). The node age of lineage I was determined to be 1960 and comprised only two historical isolates that were sampled in Senegal (1989) and Cote d’Ivoire (1989). The node age of lineage II was estimated to be around 1957 and contained nine sequences; the most recent one was collected from Liberia during the year 2015. Lineages I and II appear to have retained a West African focus of infection, despite the emergence and spread of other lineages. Lineage III included seven isolates and the node age of this group was determined to be 1965, with the most recent one being sampled in 2018, from Tanzania. Lineage IV, responsible for most of the recent PPR outbreaks, comprised 85 isolates and the node age for this lineage was estimated to be around 1967. Lineage IV was first reported from an outbreak encountered in India in 1989 [27]. The estimates of TMRCA should be interpreted cautiously, as substantial gaps in the available sequences and strong purifying selection on the PPRV genome might bias the results, as reported earlier [19].

The estimated mean for the substitution rate of the PPRV full genome was found to be 7.224 × 10^−4^ (95% HPD interval 4.088 × 10^−4^–1.065 × 10^−3^) substitutions/site/year (Table 2). The rate estimated here is slightly lower than the rate estimated earlier by Clarke and colleagues (9.22 × 10^−4^ 95% HPD 6.206 ×10^−4^–1.26 × 10^−3^) [19], Muniraju and colleagues (9.09 ×10^−4^ 95% HPD 2.13 × 10^−4^–1.64 ×10^−3^) [14], and Sahu and colleagues (7.684 × 10^−4^, 95% HPD 7.233 × 10^−4^–8.1327 × 10^−4^) [27]. The mutation frequencies in RNA viruses typically range between 10^−3^ and 10^−6^ per site per replication because of the error-prone replication of RNA polymerase and the lack of proofreading mechanisms. One of the reasons for PPRV’s ability to emerge and adapt to different geographic regions and hosts could be its greater evolutionary rate [14].

**Figure 2:**
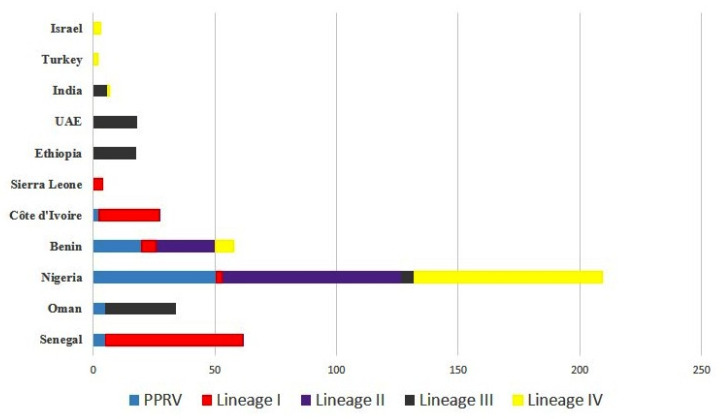
Root state posterior probabilities of PPRV and lineages I−IV based on the whole genome sequences of 103 PPRVs collected during 1969–2018.

The root state posterior probabilities (RSSP) of PPRV ranged from 0.31% to 50.32%. Nigeria (50.32%) received the highest marginal support for the whole PPRV data set (Figure 2). Analysis of the data gave Cote d’Ivorie, where PPRV was first reported, an RSSP of 2.22%. An earlier analysis suggested that Nigeria was the country of origin for PPRV, based on partial N gene sequence analysis [14]. In contrast, the data analyzed and interpreted by Benfield and colleagues [17] suggested that Benin was another potential location for the evolutionary emergence of PPRV. In the current analysis, the data gave moderate support to the possibility that Benin was the origin of the virus (19.47%). In contrast, Senegal had good root state probability support for lineage I, as suggested by Muniraju and colleagues [14], and for lineage II, Nigeria received good root state probability support in our analysis. The RSSP for lineage III could not be defined, as Ethiopia, Oman, and the United Arab Emirates all generated similar support. Benfield and colleagues [17] inferred Nigeria as the country of origin for lineage IV, and Nigeria obtained strong support in our study as well. Interestingly, despite some reports that suggest that lineage IV was initially reported in India, India’s root state marginal probability is quite low. Certainly, while the origin of lineage IV emergence remains contentious, the detection of the virus on the African continent in Cameroon in 1997 and the surrounding areas over the following years demonstrates the detection of this virus in central Africa at a time when its introduction from India must be considered unlikely. This lends support to the analysis undertaken here and elsewhere [14,17].

**Figure 3:**
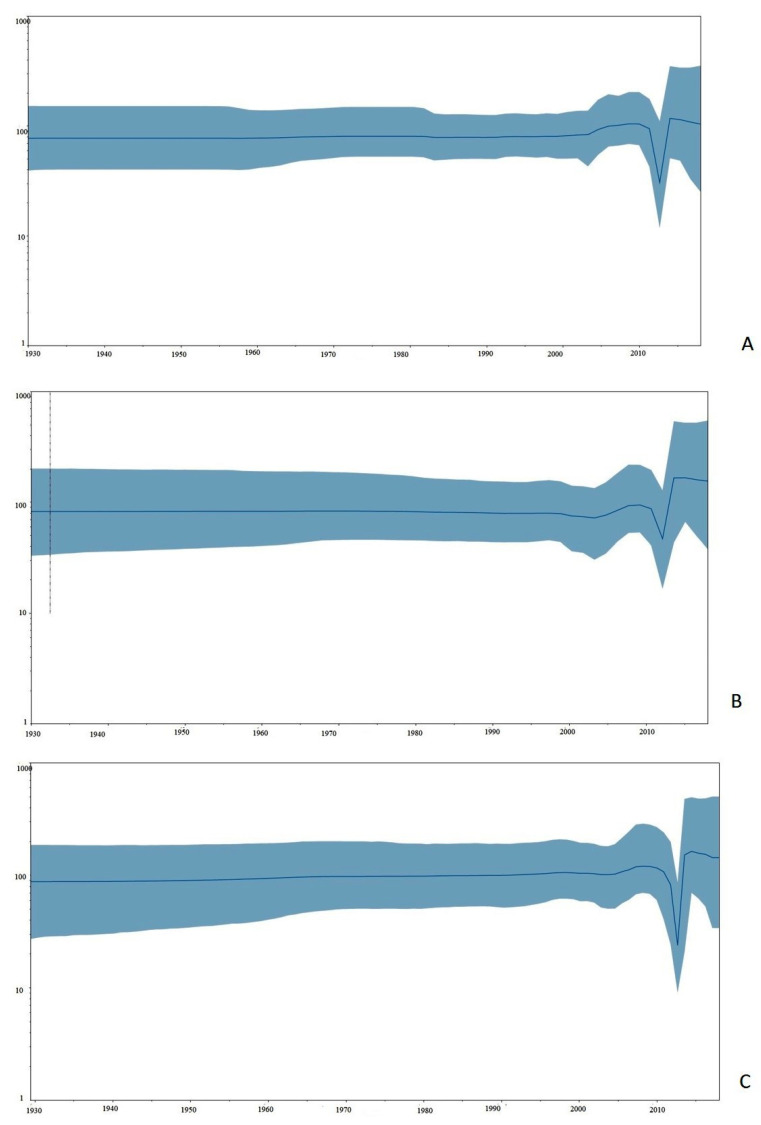
Bayesian Skyline plots (BSP) depicting temporal changes in the relative genetic diversity of PPRV isolates (*n* = 103) collected over a period of five decades (1969–2018) across the globe. Line plots summarize estimates of the effective population size (*y*-axis) for full genome and the shaded regions correspond to the 95% HPD. (**A**): Bayesian skyline plot estimated by strict molecular clock. (**B**): Bayesian skyline plot estimated by uncorrelated exponential deviation clock (UCED). (**C**): Bayesian skyline plot estimated by uncorrelated log-normal clock (UCLN).

The effective population size of PPRV remained stable until 2010, according to a coalescence-based Bayesian skyline map (Figure 3). The population decreased substantially between 2011 and 2012, followed by a period of population rise around 2013, and then a period of stability since 2015. The pan-China PPR epizootic during 2013–2014 may have justified the calculated population growth [28], but the source of the sudden drop in population size is uncertain, which may be ascribed to the initiation of PPR vaccination around the globe. Furthermore, the circulation of predominantly three PPRV lineages after 2013, following the apparent disappearance of lineage I, since 1989, may have aided the attainment of the maximum effective population. Interestingly, more recent reports, from regions not yet well studied, have demonstrated the ongoing circulation of lineage I in West Africa [4], although full genome analyses have not been available to enable the inclusion of this finding within evolutionary assessments. Future analysis of PPR across West Africa ruminant populations may yield further samples to enable a better evolutionary assessment of lineage I viruses. Throughout the 1990s, the utilization of the RPV vaccine to protect sheep and goats against PPRV may have driven the virus down a genetic bottleneck that may reflect its long-term stable genetic diversity [14]. However, since the cessation of RPV vaccine administration during the eradication of RPV, the epidemiology and genetic diversity observed in the case of PPRV have become more complex.

The AI and PS statistics are used to measure the strength of phylogenetic clustering by traits, as well as the significance of the *p*-value (0.05). The index ratio (IR) of zero implies complete population subdivision, whereas a value of one suggests random mixing. The null hypothesis of no link between the geographic and phylogenetic relationship was rejected by the global trait association analyses of phylogeographic structure. The IR of the observed values, compared to those expected of AI (0.31) and PS (0.47), for the PPR virus full genome demonstrates that evolution is not homogenous, but rather presents a geographic structure (Table 3). When the MC statistic was displayed, this trait became more apparent. The population subdivision was significant for many countries, including Israel, China, India, Pakistan, Ethiopia, and Mongolia. This shows that population differentiation among PPRV populations is influenced by location.

The evolutionary rate of the F gene has been determined to be much faster than that of other genes, as well as the entire genome (Table 4). The strongest evolutionary correlation coefficient was found in the F gene, followed by M, P, L, N, and H, in that order. All of the genes had frequent alterations at the third codon position, followed by the first codon position, and the second codon position, which, as expected, was found to be generally well conserved. In comparison to other genes, relative substitution at the third codon was found to be low for the P gene, while substitution at codon positions 1 and 2 was found to be higher. Overall, the F gene may be the greatest candidate for evolutionary and phylogenetic study.

## 4. Conclusions

In conclusion, wherever new full genome sequences are defined, further utilization of evolutionary analyses is critical in enabling re-assessment of the genetic diversity and evolution of PPRV. However, whilst data sets continue to be made available, significant gaps remain in the knowledge of circulating viruses over vast time periods and geographical areas. The attempts of this study and previous studies to fill the gaps in knowledge around wildlife sequences are important, but the fundamental linkage to domestic animals is likely a better source for new sequences as they arise, since outbreaks can be contained and, hence, more easily sampled. Mass mortality events in wildlife likely offer the only opportunity to sample these disparate and largely inaccessible populations. This fundamental limitation means that such analyses should be interpreted with caution, and that reassessment with new genome data is critical to further understand the molecular evolution of the virus. As stated, the bias of the sample types towards domesticated livestock, where outbreaks of PPR are more frequently reported, means that the genetic diversity across domesticated versus wild animal populations cannot be rigorously assessed. This paucity in comparative data has been recognized in other reports in this area of computational evolutionary biology. Regardless, these analyses help shape our understanding of the complex molecular epidemiology of the disease, and enable factors that may have shaped viral evolution, including host switches and vaccination strategies, to be assessed.

## Figures and Tables

**Table 1:** TMRCA estimate of PPRV and its different lineages.

Data Set	Time Period	Mean Evolutionary Rate	95% HPD Interval		Reference
			Lower	Upper	
12	1976–2012	9.09 × 10^−4^	2.13 × 10^−4^	1.64 × 10^−3^	Muniraju et al., 2014
27	1969–2011	7.8 × 10^−4^	7.3 × 10^−4^	8.4 × 10^−4^	Adombi et al., 2015
37	1969–2015	7.684 ×10^−^^4^	7.233 ×10^−^^4^	8.1327 ×10^−^^4^	Sahu et al., 2017
37	1969–2014	9.22 × 10^−4^	6.206 × 0^−4^	1.26 × 10^−3^	Clarke et al., 2017
81	1969–2018	9.22 × 10^−4^	6.78 × 0^−4^	1.17 × 10^−3^	Benfield et al., 2021
103	1969–2018	8.098 × 10^−4^	5.867 × 10^−4^	9.056 × 10^−4^	This study

**Table 2:** Estimate of mean evolutionary rates of PPRV. UCED: uncorrelated exponential distribution; CS: constant size; EG: exponential growth.

Set Description	Root Age	95% HPD Interval		Number of Sequences	Time Period
		Lower	Upper		
PPRV-Common Ancestor	1904	1715	1967	103	1969–2018
Common ancestor of lineage I and III	1924	-	-	9	1969–2018
Common ancestor of lineage II and IV	1909	1936	1954	94	1969–2018
Lineage I	1960	1940	1968	2	1969–1989
Lineage II	1957	1943	1965	9	1969–2015
Lineage III	1965	1944	1977	7	1983–2018
Lineage IV	1967	1948	1981	85	1994–2018

**Table 3:** Phylogeny trait association tests of the phylogeographic structure of PPRV using Bayesian Tip-association Significance testing (BaTS).

Statistics	Observed Mean (95% CI)	Null Mean (95% CI)	*p*-Value
AI	2.78 (2.77, 2.79)	9.09 (8.26, 9.82)	0.00
PS	29.0 (29.0, 29.0)	61.66 (59.5, 64.0)	0.00
MC (India)	6.0 (6.0, 6.0)	1.23 (1.0, 2.0)	0.009
MC (Israel)	18.0 (18.0, 18.0)	1.81 (1.0, 3.0)	0.009
MC (China)	14.5 (11.0, 18.0)	2.75 (2.0, 4.0)	0.009
MC (Pakistan)	3.0 (3.0, 3.0)	1.0 (1.0,1.0)	0.009
MC (Ethiopia)	2.0 (2.0, 2.0)	1.0 (1.0,1.0)	0.029
MC (Mongolia)	4.0 (4.0, 4.0)	1.0 (1.0,1.0)	0.029

**Table 4:** Evolutionary parameters of different genes of PPRV.

Gene	Evolutionary Rate (95% HPD)	Evolutionary Correlation Coefficients (95% HPD)	Relative Substitution Rate of Different Codon Position		
			1	2	3
N	6.547 × 10^−4^ (4.691–8.444 × 10^−4^)	0.917 (0.8139–1.0231)	0.474 (0.367–0.586)	0.283 (0.214–0.356)	2.243 (2.119–2.361)
P	6.33 × 10^−4^ (4.541–8.233 × 10^−4^)	0.952 (0.8477–1.0538)	0.83 (0.712–0.952)	0.675 (0.574–0.779)	1.494 (1.365–1.619)
M	6.753 × 10^−4^ (4.681–8.89 × 10^−4^)	0.975 (0.866–1.0822)	0.571 (0.387–0.764)	0.145 (0.089–0.203)	2.284 (2.103–2.478)
F	3.419 × 10^−3^ (2.307–4.657 × 10^−3^)	1.069 (0.964–1.1745)	0.449 (0.382–0.519)	0.312 (0.257–0.369)	2.230 (2.152–2.325)
H	6.37 × 10^−4^ (4.561–8.227 × 10^−4^)	0.914 (0.815–1.0206)	0.542 (0.461–0.625)	0.433 (0.359–0.51)	2.026 (1.924–2.122)
L	5.071 × 10^−4^ (3.681–6.446 × 10^−4^)	0.921 (0.8178–1.0217)	0.451 (0.405–0.497)	0.229 (0.196–0.264)	2.32 (2.266–2.373)

## Data Availability

The sequence data sets generated during this research are publicly available at NCBI GenBank.

## Supplementary Materials

The following are available online at https://www.mdpi.com/article/10.3390/v13112144/s1: Table S1: title list of PPRV genomes used in this study.

## References

[1] Banyard A.C., Parida S., Batten C., Oura C., Kwiatek O., Libeau G. (2010). Global distribution of peste des petits ruminants virus and prospects for improved diagnosis and control. J. Gen. Virol..

[2] Parida S., Muniraju M., Mahapatra M., Muthuchelvan D., Buczkowski H., Banyard A.C. (2015). Peste des petits ruminants. Vet. Microbiol..

[3] Kumar K.S., Babu A., Sundarapandian G., Roy P., Thangavelu A., Kumar K.S., Arumugam R., Chandran N.D., Muniraju M., Mahapatra M. (2014). Molecular characterisation of lineage IV peste des petits ruminants virus using multi gene sequence data. Vet. Microbiol..

[4] Tounkara K., Kwiatek O., Sidibe C.A.K., Sery A., Dakouo M., Salami H., Lo M.M., Ba A., Diop M., Niang M. (2021). Persistence of the historical lineage I of West Africa against the ongoing spread of the Asian lineage of peste des petits ruminants virus. Transbound. Emerg. Dis..

[5] Mariner J.C., House J.A., Mebus C.A., Sollod A.E., Chibeu D., Jones B.A., Roeder P.L., Admassu B., van’t Klooster G.G. (2012). Rinderpest eradication: Appropriate technology and social innovations. Science.

[6] Mitchell D.T., Le R.P. (1946). Further investigations into immunization of cattle against rinderpest. Onderstepoort. J. Vet. Sci. Anim. Ind..

[7] (1946). VETERINARY CORPS develops a new rinderpest vaccine. Vet. Med..

[8] Zhao H., Njeumi F., Parida S., Benfield C.T.O. (2021). Progress towards Eradication of Peste des Petits Ruminants through Vaccination. Viruses.

[9] Furley C.W., Taylor W.P., Obi T.U. (1987). An outbreak of peste des petits ruminants in a zoological collection. Vet. Rec..

[10] Li L., Cao X., Wu J., Dou Y., Meng X., Liu D., Liu Y., Shang Y., Liu X. (2019). Epidemic and evolutionary characteristics of peste des petits ruminants virus infecting Procapra przewalskii in Western China. Infect. Genet. Evol..

[11] Pruvot M., Fine A.E., Hollinger C., Strindberg S., Damdinjav B., Buuveibaatar B., Chimeddorj B., Bayandonoi G., Khishgee B., Sandag B. (2020). Outbreak of Peste des Petits Ruminants among Critically Endangered Mongolian Saiga and Other Wild Ungulates, Mongolia, 2016–2017. Emerg. Infect. Dis..

[12] Aguilar X.F., Fine A.E., Pruvot M., Njeumi F., Walzer C., Kock R., Shiilegdamba E. (2018). PPR virus threatens wildlife conservation. Science.

[13] Bailey D., Banyard A., Dash P., Ozkul A., Barrett T. (2005). Full genome sequence of peste des petits ruminants virus, a member of the Morbillivirus genus. Virus Res..

[14] Muniraju M., Munir M., Parthiban A.R., Banyard A.C., Bao J., Wang Z., Ayebazibwe C., Ayelet G., El Harrak M., Mahapatra M. (2014). Molecular Evolution of Peste des Petits Ruminants Virus. Emerg. Infect. Dis..

[15] Diallo A., Taylor W.P., Lefevre P.C., Provost A. (1989). Attenuation of a strain of rinderpest virus: Potential homologous live vaccine. Rev. Elev. Med. Vet. Pays. Trop..

[16] Adombi C.M., Waqas A., Dundon W.G., Li S., Daojin Y., Kakpo L., Aplogan G.L., Diop M., Lo M.M., Silber R. (2017). Peste Des Petits Ruminants in Benin: Persistence of a Single Virus Genotype in the Country for Over 42 Years. Transbound. Emerg. Dis..

[17] Benfield C.T.O., Hill S., Shatar M., Shiilegdamba E., Damdinjav B., Fine A., Willett B., Kock R., Bataille A. (2021). Molecular epidemiology of peste des petits ruminants virus emergence in critically endangered Mongolian saiga antelope and other wild ungulates. Virus Evol..

[18] Masdooq A.A., Pawar R.M., Parthiban A.B., Ragavendhar K., Sundarapandian G., Thangavelu A., Dhinakar Raj G. (2015). Complete Genome Sequences of Lineage IV Peste des Petits Ruminants Viruses from the Indian Subcontinent. Genome Announc..

[19] Clarke B., Mahapatra M., Friedgut O., Bumbarov V., Parida S. (2017). Persistence of Lineage IV Peste-des-petits ruminants virus within Israel since 1993: An evolutionary perspective. PLoS ONE.

[20] Darriba D., Taboada G.L., Doallo R., Posada D. (2012). jModelTest 2: More models, new heuristics and parallel computing. Nat. Methods.

[21] Rambaut A., Lam T.T., Max Carvalho L., Pybus O.G. (2016). Exploring the temporal structure of heterochronous sequences using TempEst (formerly Path-O-Gen). Virus Evol..

[22] Suchard M.A., Lemey P., Baele G., Ayres D.L., Drummond A.J., Rambaut A. (2018). Bayesian phylogenetic and phylodynamic data integration using BEAST 1.10. Virus Evol..

[23] Baele G., Lemey P., Bedford T., Rambaut A., Suchard M.A., Alekseyenko A.V. (2012). Improving the accuracy of demographic and molecular clock model comparison while accommodating phylogenetic uncertainty. Mol. Biol. Evol..

[24] Parker J., Rambaut A., Pybus O.G. (2008). Correlating viral phenotypes with phylogeny: Accounting for phylogenetic uncertainty. Infect. Genet. Evol..

[25] Ronquist F., Huelsenbeck J.P. (2003). MrBayes 3: Bayesian phylogenetic inference under mixed models. Bioinformatics.

[26] Baazizi R., Mahapatra M., Clarke B.D., Ait-Oudhia K., Khelef D., Parida S. (2017). Peste des petits ruminants (PPR): A neglected tropical disease in Maghreb region of North Africa and its threat to Europe. PLoS ONE.

[27] Sahu A., Wani S., Saminathan M., Rajak K., Sahoo A., Pandey A., Saxena S., Kanchan S., Mishra B., Muthuchelvan D. (2017). Genome sequencing of an Indian peste des petits ruminants virus isolate, Izatnagar/94, and its implications for virus diversity, divergence and phylogeography. Arch. Virol..

[28] Bao J., Wang Q., Li L., Liu C., Zhang Z., Li J., Wang S., Wu X., Wang Z. (2017). Evolutionary dynamics of recent peste des petits ruminants virus epidemic in China during 2013–2014. Virology.

